# Electric Field Dependence of EPR Hyperfine Coupling Constants

**DOI:** 10.1021/acs.jpca.4c04480

**Published:** 2024-09-12

**Authors:** Tadeusz Pluta, Grzegorz Skrzyński

**Affiliations:** Institute of Chemistry, University of Silesia in Katowice, Szkolna 9, 40-006 Katowice, Poland

## Abstract

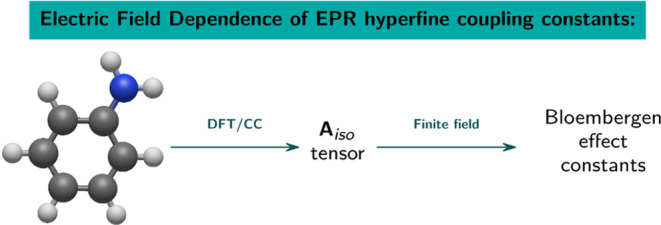

In this article, we introduce a simple but reliable method to calculate the electric field dependence of the isotropic hyperfine coupling tensor **A**_iso_ for free radicals. This dependence, also referred to as the Bloembergen effect, can be of interest in analyzing EPR experiments for solid-state materials but is rarely studied for isolated radicals in the gaseous phase. The proposed method uses the numerical differentiation of the field-perturbed **A** tensor and, consequently, as a purely numerical method, does not depend on a quantum chemical method used to determine the hyperfine tensor **A**. To test the performance of the proposed method, we used a set of 28 systems, including seven organic radicals, the majority of which were taken from a recent benchmark study by Bartlett’s group. We employed the well-tested and robust density functional theory (DFT) functional, namely CAM-B3LYP, and the variant of the CCSD method based on local pair natural orbitals, namely DLPNO-CCSD to calculate the **A**_iso_ tensor itself, and all first derivatives or Bloembergen effect constants *a_i_*^(1)^.

## Introduction

The interaction between electron spin magnetic moments and nuclear magnetic moments gives rise to the hyperfine structure of EPR spectra. It is conveniently described as the hyperfine coupling tensor **A**. The hyperfine part of the spin-Hamiltonian is expressed by the general formula^1^
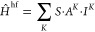
1The hyperfine coupling tensor for the *K*-th nucleus **A**^*K*^ is composed of two terms: the isotropic or Fermi contact (FC) term and the anisotropic spin-dipolar term. The isotropic FC **A**_iso_ term is usually larger and often dominates the hyperfine interactions. Typically, **A**_iso_ is expressed in terms of the Dirac delta function, which makes this property directly proportional to the electron spin density at the nucleus. The isotropic component of the hyperfine coupling tensor **A** for the nucleus *K* can be calculated as the expectation value of the following operator
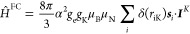
2where α is the fine structure constant, *g*_e_/*g*_K_ are the electron and nuclear g factors, μ_B_/μ_N_ are the Bohr and nuclear magnetic constants, *r*_iK_ is the electron *i* – nucleus *K* distance, and ***s***_**i**_ and ***I***^K^ denote the electron and nuclear spin operators, respectively.^2^ Spin–orbit effects have also been shown to contribute to the hyperfine coupling constants. However, we deliberately confined our study to a set of light organic radicals for which spin–orbit corrections are likely to be small. Theoretical calculations of the hyperfine coupling constant have been the subject of many theoretical studies for the reviews; see refs (3) and (4). The overall conclusion that can be drawn from these calculations is the necessity of using accurate quantum chemical methods, like Coupled Cluster (CC), to obtain accurate values of the **A** tensor, e.g., ref (5). However, the high numerical cost of the CCSD (CC with Singles and Doubles)^6^ calculations makes the usage of this method for larger systems still a challenge. Density functional theory (DFT) methods, with their favorable cost/accuracy factor, have become a method of choice for theoretical studies on the hyperfine tensor **A**, e.g., review.^7^ Despite the growing number of functionals, there is no systematic and theoretically justified method to increase the accuracy of the DFT calculations for the hyperfine coupling constant. Therefore, the best way to validate the results is to compare results obtained with different DFT functionals to more reliable CCSD values, if available, e.g., refs (8−10).

**Table I:** Isotropic Hyperfine Coupling Tensor **A**_iso_ (in MHz) and its First Derivatives – Bloembergen Effects Constants *a_i_*^(1)^ (in 10^11^·G·m/V) of the SiH_3_ and OH Radicals

system and point group	nuclei	**A**_iso_	*a_x_*^(1)^	*a_y_*^(1)^	*a_z_*^(1)^	**A**_iso_	*a_x_*^(1)^	*a_y_*^(1)^	*a_z_*^(1)^	**A**_iso_	A_iso_	*a_x_*^(1)^	*a_y_*^(1)^	*a_z_*^(1)^	**A**_iso_
		DLPNO-CCSD				CAM-B3LYP				CCSD^a^^11^	Karna^b^^19,20^				Exp.^c^
SiH_3_	^29^Si	–507.80			–629.3	–481.54			–651.6		–490.10			–596.62	
*C*_3*v*_	^1^H	18.99	–19.2		–57.9	36.23	–16.5		–66.0		21.13	–47.32		–67.51	
	^1^H	18.99	9.6	–16.6	–57.9	36.23	8.3	–14.3	–66.0		21.13	23.66	–40.98	–67.51	
	^1^H	18.99	9.6	16.6	–57.9	36.23	8.3	14.3	–66.0		21.13	23.66	40.98	–67.51	
OH	^17^O	–44.84	–17.2			–39.03	–14.9			–50.9	–84.92	–28.43			51.3
*C*_∞*v*_	^1^H	–77.56	2.4			–66.93	2.6			–77.1	–124.91	18.45			71.5

a aug-cc-pVTZ-J basis set was used in ref (11).

b For SiH_3_, the PUHF/DZP and TDUHF/DZP results are taken from ref (19), and for OH, the TDUHF/Pol results are taken from ref (20).

c Experimental results come from ref (11). The source of these values is provided therein. Experimental values are unsigned.

**Figure 1:**
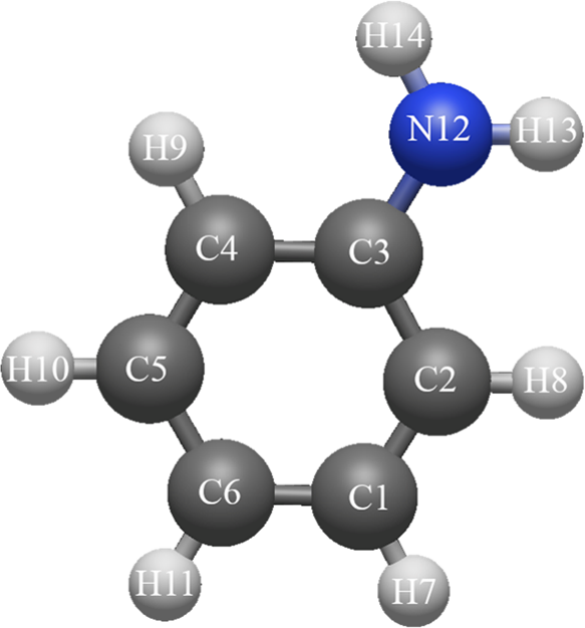
Structure of the aniline^+^ cation with atom labels.

**Table II:** Isotropic Hyperfine Coupling Tensor **A**_iso_ (in MHz) and its First Derivatives – Bloembergen Effects Constants *a_i_*^(1)^ (in 10^11^·G·m/V) of the Aniline^+^ Cation

system and point group	nuclei	**A**_iso_	*a_x_*^(1)^	*a_y_*^(1)^	*a_z_*^(1)^	**A**_iso_	*a_x_*^(1)^	*a_y_*^(1)^	*a_z_*^(1)^	**A**_iso_
		DLPNO-CCSD				CAM-B3LYP				CCSD/aug-cc-pVTZ-J^11^
aniline^+^	^13^C1, ^13^C5	–26.50	–23.0	3.8; −3.0	–1.2; −1.3	–22.47	–20.5	4.8; −4.1	–0.9; −1.1	–25.2
*C*_2*v*_	^13^C2, ^13^C4	16.33	44.2	–3.2; 1.6	2.4; 2.5	13.27	35.4	–3.3; 2.1	1.8; 1.9	12.5
	^13^C3	–19.31	–79.8	1.4	–4.3	–17.13	–65.3	1.2	–3.5	–19.4
	^13^C6	36.89	12.7	–0.2	0.7	32.57	10.8	–0.2	0.4	35.2
	^1^H7, ^1^H10	4.93	17.5; 17.8	–8.5; 7.9	0.7; 1.2	6.29	17.1; 17.4	–8.7; 8.1	0.6; 1.1	4.0
	^1^H8, ^1^H9	–16.85	–19.9; −20.0	2.3; −1.6	–1.0; −1.1	–16.04	–19.7; −19.7	1.8; −1.1	–1.0; −1.0	–15.8
	^1^H11	–34.19	10.1	–0.2	0.5	–30.63	8.6	–0.2	0.6	–33.8
	^1^H13, ^1^H14	–30.62	–39.3	0.5; 0.9	–2.2	–27.13	–35.4	0.8; 0.5	–2.0	–28.7
	^14^N12	17.96	26.7	–0.5	1.5	14.57	21.5	–0.4	1.2	17.2

In the benchmark study of Bartlett and his co-workers,^11^ the detailed analysis of the performance of ‘gold standard’ CCSD(T) (CCSD plus noniterative triples) and CCSD methods for the set of 55 radicals was presented and compared to DFT results. None of the investigated functionals stood up as ‘the best functional’ for the purpose, while CAM-B3LYP,^12^ CAM-QTP01, and CAM-QTP02^13^ were competitive with CC results as shown by the values of the root-mean-square error for two first subsets of the studied radicals, for details see ref (11). The choice of the proper basis set for calculations of the **A**_iso_ tensor has also been paid considerable attention. The dependence of the FC contribution on the electron spin density at the position of the nucleus suggests a better description of the core region than provided by standard bases. Therefore, suitable basis sets should contain additional, optimized tight functions. The often used bases of this type are EPR-II/III sets of Barone.^14^ Recently, Jakobsen and Jensen^15^ developed a new series of basis sets suitable for the hyperfine coupling tensor calculations called pcH-n. It was also noticed that the augmented polarization aug-cc-PVTZ-J^16^ basis developed for the nuclear spin–spin coupling works very well for the hyperfine coupling calculations.

Computation of **A** tensor can provide a useful and interesting comparison for a variety of advanced theoretical methods, but it is also a valuable tool to elucidate experimental results provided by EPR spectroscopy. To analyze EPR experiments, one has to take into account many factors like external electric fields, solvent effects, crystal lattice, etc. Our study is concerned with the presence of the external electric field ***F*** and its effect on the **A**_iso_ tensor. Electric field dependence of the hyperfine coupling constant was predicted by Bloembergen^17^ and studied for many solid-state radical systems, e.g., see the monograph by Mims.^18^ However, the effect has attracted little interest in the quantum chemistry community. Karna used perturbation theory to develop a theory of the Bloembergen effect and calculated the dependence of **A** on the electric field for a few small radicals like SiH_3_ and OH.^19,20^ Since for the SiH_3_ radical, the author used the Unrestricted Hartree–Fock (UHF) theory and modest basis sets of DZP quality,^19^ his results may be treated as reasonable estimates only. The same level of theory and the basis set of Sadlej^21^ was used in the study of the OH radical.^20^

Our goal is to introduce a straightforward method to calculate the first-order derivatives of the **A**_iso_, or the Bloembergen effect constants,^19^ with respect to the components of the external electric field using a numerical differentiation technique, also referred to as the finite-field approach. The greatest advantage of the numerical approach is that it does not depend on the electronic structure method.

We chose to employ the domain-based local pair natural orbital coupled cluster singles and doubles (DLPNO-CCSD) method developed by Neese and his co-workers.^22,23^ The method was implemented and applied for accurate calculations of spin densities.^24^ We also included the CAM-B3LYP functional^12^ in our computations. The recent study of **A**_iso_ of Bartlett et al.^11^ showed good accuracy of the CAM-B3LYP results when compared to the benchmark CCSD(T) and CCSD values. Since our study is concerned with the derivatives of the **A**_iso_ tensor, not the value of the tensor itself, we do not comment on the performance of various DFT functionals in comparison to advanced *ab initio* methods like Coupled Cluster. The comprehensive recent study^11^ serves as an excellent source of relevant data.

We selected a group of twenty-one small radicals; nineteen of them are the subset I studied by Bartlett. We added the two small radicals, SiH_3_ and CN, and included a group of seven larger organic radicals, also considered by Bartlett et al.^11^

The paper is organized as follows: in the next section, we briefly describe the computational details of our approach, and then we present our results. The summary is given in the last section.

## Methods

All calculations were done using ORCA program version 5.0.3.^25,26^ installed on a Linux workstation. To facilitate the comparison of the results of **A**_iso_ from Bartlett et al.,^11^ we used the same geometry without modifications or changing labels of molecular axes and the same basis set, aug-cc-pVTZ-J.^16^ We studied nineteen systems from the small radicals subset of ref (11) together with the two added radicals and seven larger organic radicals: the aniline cation, the 4-nitroaniline cation, benzyl, 1,3,2-benzodithiazolyl, 1-adamantyl, phenylaminyl, and cyclo-hexyl. SiH_3_ radical with the geometry taken from Karna^19^ and CN radical with the bond length taken from NIST^27^ were added to the studied set of small radicals. For the larger organic radicals, with the exception of the aniline cation, we performed only CAM-B3LYP^12^ calculations. As can be seen in Bartlett et al. results,^11^ the CAM-B3LYP values of **A**_iso_ for these systems are in good agreement with theoretically more advanced and more expensive CCSD results. Static external electric field of the strength 0.005 au (2.5711034·10^9^ V/m) was used in all calculations. DLPNO-CCSD calculations were performed with the tightening truncation settings (Default 2) recommended in^24^ to ensure accurate values.

The numerical or finite-field technique has been frequently used to calculate molecular properties that can be expressed as partial derivatives of energy with respect to an external perturbation. The simple way to realize the required differentiation is to expand the perturbed property, **A** in our case, in the Taylor-like series
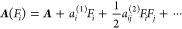
3
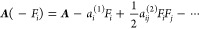
4where *F_i_* is the *i-*th component of the external electric field ***F***, and *a_i_*^(1)^ is the desired first-order derivative or the Bloembergen effect coefficient. Subtracting ***A***(−***F***) from ***A***(***F***), we get a simple expression for the *a_i_*^(1)^ constants. To eliminate contamination introduced by the next power of the external field (of the same parity), one can set up the analogous pair to eqs 3 and 4 but for the field with twice the initial strength, 2*F*. The simple manipulation of the two pairs yields the first derivatives, contaminated only by the fifth-order terms
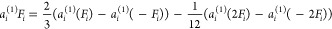
5Bartlett and Purvis^28^ presented a set of formulas for the higher-order electric properties using two values of an external electric field. In this study, we are concerned only with the first derivatives of **A**, or Bloembergen effect constants, and the pair of eqs 3 and 4 were found sufficient to obtain the reported numerical precision.

## Results and Discussion

Table I contains the values of **A**_iso_ and its first derivatives of the SiH_3_ and OH radicals. The pyramidal geometry of the SiH_3_ structure was taken without modifications from Karna’s paper.^19^ The units of *a_i_*^(1)^ (*i* = *x*, *y*, or *z*) are G·m/V as used by Karna in ref (19).

**Table III:** Calculated Values of the Isotropic Fermi Contact **A**_iso_ Tensor (in MHz) and the First-order Derivatives (Bloembergen Effect Constants) *a_i_*^(1)^ (in 10^11^·G·m/V)

system and point group	nuclei	**A**_iso_	*a_x_*^(1)^	*a_y_*^(1)^	*a_z_*^(1)^	**A**_iso_	*a_x_*^(1)^	*a_y_*^(1)^	*a_z_*^(1)^	**A**_iso_	**A**_iso_
		DLPNO-CCSD				CAM-B3LYP				Theor.^a^	Exp.^b^
H_2_O^+^	^17^O	–75.81	–5.3	–7.5		–60.68	–4.7	–6.6		–81.2	83.2
*C*_2*v*_	^1^H	–80.44	7.9	–1.0		–71.06	7.0	–0.8		–80.1	73.1
	^1^H	–80.44	–3.5	7.1		–71.06	–3.1	6.4		–80.1	73.1
SH	^33^S	26.00	17.2			8.17	18.0			34.5	
*C*_∞*v*_	^1^H	–56.18	–3.5			–38.67	–4.4			–53.7	65.0
HCS	^13^C	287.87	–38.3		273.8	288.56	–46.5		269.3	294.0	
*C_s_*	^1^H	119.07	–42.1		–81.8	133.82	–39.3		–81.7	123.1	127.5
	^33^S	14.12	–32.5		5.0	9.35	–27.5		23.8	18.6	
CO^+^	^13^C	1529.90	–528.7			1630.10	–522.2			1596.0	1573.0
*C*_∞*v*_	^17^O	34.31	49.7			27.77	56.7			23.4	18.5
CH	^1^H	–62.38	–9.1			–49.82	–9.0			–60.3	57.7
*C*_∞*v*_	^13^C	30.11	31.9			43.79	32.3			40.8	47.1
CH_2_	^13^C	233.71			–157.7	228.26			–162.0	239.7	
*C*_2*v*_	^1^H	–23.83	12.0		22.4	–10.89	12.5		25.0	–22.9	20.2
	^1^H	–23.83	–12.0		22.4	–10.89	–12.5		25.0	–22.9	20.2
CH_2_^–^	^13^C	55.54			190.4	68.64			203.4	68.2	58.9
*C*_2*v*_	^1^H	–48.77	5.7		–16.7	–39.48	3.4		–11.1	–46.2	44.8
	^1^H	–48.77	–5.7		–16.7	–39.48	–3.4		–11.1	–46.2	44.8
C_2_H	^13^C	225.50	–59.9			236.75	–60.5			224.7	213.0
*C*_∞*v*_	^13^C	1029.79	–404.3			1068.10	–408.6			1049.7	1014.5
	^1^H	57.09	15.6			56.49	14.9			54.8	50.4
CH_3_	^13^C	70.46				73.89				77.2	75.7
*D*_3*h*_	^1^H	–76.49	–2.9	5.0	–1.7	–65.49	–2.7	4.7	–1.6	–74.0	70.1
	^1^H	–76.49	–2.8	–4.0	3.5	–65.49	–2.6	–3.8	3.3	–74.0	70.1
	^1^H	–76.49	5.7	–1.0	–1.8	–65.49	5.3	–0.9	–1.6	–74.0	70.1
CH_2_CH	^13^C	–20.74	–9.2	–25.4		–12.48	9.6	–27.3		–17.9	24.0
*C_s_*	^13^C	317.19	116.4	–220.4		313.53	107.6	–226.6		322.4	301.5
	^1^H	166.39	–39.7	9.8		175.94	–44.8	12.6		161.4	192.0
	^1^H_CH_	100.91	–21.4	4.4		111.80	–18.4	2.6		96.2	95.8
	^1^H	36.15	–74.6	34.6		54.33	–80.1	40.0		37.9	37.3
HOO	^1^H	–19.72	–9.3		59.2	–20.99	–9.0		–10.3	–22.9	27.4
*C_s_*	^17^O	–60.28	–13.0		–14.1	–42.91	–7.0		–8.2	–61.1	
	^17^O	–29.36	–19.8		–20.3	–26.74	–15.1		–14.2	–34.8	
HCO	^13^C	375.14	–216.3	–295.7		384.30	–230.9	–293.0		388.4	377.5
*C_s_*	^1^H	363.85	–10.4	88.6		380.26	2.7	86.8		360.0	354.0
	^17^O	–42.17	9.1	5.7		–36.30	9.5	13.6		–44.4	42.3
H_2_CCO^+^	^13^C_CH2_	68.70	–13.0	–24.7		61.71	–9.3	–17.6		67.3	
*C*_2*v*_	^13^C_CO_	–69.84	11.8	22.5		–56.20	12.9	24.4		–69.7	
	^1^H	–68.68	6.0	13.4		–59.44	5.2	10.4		–66.1	58.0
	^1^H	–68.76	7.7	12.5		–59.48	5.6	10.1		–66.1	58.0
	^17^O	–20.28	–1.2	–2.6		–15.20	–1.0	–2.0		–20.4	
NH_2_	^1^H	–72.35	–2.8	–1.3		–61.38	–2.4	–0.8		–70.4	67.2
*C*_2*v*_	^1^H	–72.35	–0.2	–3.0		–61.38	0.1	–2.5		–70.4	67.2
	^14^N	26.41	5.9	8.3		25.23	5.5	7.8		28.3	27.9
NH_3_^+^	^14^N	44.76				39.27				47.1	54.9
*D*_3*h*_	^1^H	–87.16		–3.3		–76.12		–2.4		–85.5	76.8
	^1^H	–87.16		1.7	2.9	–76.12		1.2	2.1	–85.5	76.8
	^1^H	–87.16		1.7	–2.9	–76.12		1.2	–2.1	–85.5	76.8
CN	^13^C	556.52			–992.5	627.88			–1081.9	626.4^c^,	588.5
										581.2^d^,	
										565.0^e^	
*C*_∞*v*_	^14^N	–24.86			29.3	–26.75			–0.9	–26.7^c^,	12.6
										–17.4^d^,	
										–21.6^e^	
H_2_CN	^13^C	–83.02	–3.5			–71.36	–2.1			–81.6	81.0
*C*_2*v*_	^1^H	219.86	–55.5	–17.7		229.66	–59.7	–13.7		212.3	233.2
	^1^H	219.86	–55.5	17.7		229.66	–59.7	13.7		212.3	233.2
	^14^N	24.98	–7.3			21.28	–6.2			26.4	25.8
H_2_CCN	^13^C	71.32	0.3	0.3		67.83	–1.5	–2.6		74.2	
*C*_2*v*_	^13^C	–74.44	2.1	3.9		–64.12	1.2	2.0		–73.4	
	^1^H	–69.25	–4.8	–2.8		–60.99	–5.1	–3.0		–67.5	58.8
	^1^H	–69.25	–0.2	–5.4		–60.99	0.0	–6.0		–67.5	58.8
	^14^N	9.76	1.2	2.4		9.07	1.3	2.2		9.8	9.8
PH_3_^+^	^31^P	1205.01	–898.8	–1.9	0.0	1062.21	–904.1	–0.2	0.1	1212.8	1176.0
*C*_3*v*_	^1^H	0.85	36.9	11.8		15.18	42.0	13.0		1.0	<5.9
	^1^H	0.85	36.9	–6.0	–10.2	15.18	42.0	–6.5	–11.3	1.0	<5.9
	^1^H	0.85	36.9	–6.0	10.2	15.18	42.0	–6.5	11.3	1.0	<5.9

a All values unmarked are CCSD/aug-cc-pVTZ-J results from ref (11).

b Experimental results for CN come from ref (29). For the rest of the systems, experimental results come from ref (11). Sources of these values are provided therein. Experimental values are unsigned.

c CAM-B3LYP/IGLO-III values from ref (10).

d DLPNO-CCSD/IGLO-III values from ref (10).

e B3LYP/cc-pCVQZ values from (30).

**Table IV:** Calculated Values of the Isotropic Fermi Contact **A**_iso_ Tensor (in MHz) and the First-order Derivatives (Bloembergen Effect Constants) *a_i_*^(1)^ (in 10^11^·G·m/V) for the Set of Selected Organic Radicals

system and point group	nuclei	**A**_iso_	*a_x_*^(1)^	*a_y_*^(1)^	*a_z_*^(1)^	**A**_iso_
		CAM-B3LYP				ref^a^
4-nitroaniline^+^	^13^C1, ^13^C5	–24.68	–20.3	5.4; −5.0	0.4	–28.0
*C*_2_	^13^C2, ^13^C4	16.41	36.4	–6.1; 5.4	–1.2; −0.3	16.4
	^13^C3	–21.15	–68.8	0.8	1.4	–24.2
	^13^C6	43.20	7.4	0.0	–0.1	48.8
	^1^H7, ^1^H10	8.61	17.2	–7.6; 7.3	–0.3	6.5
	^1^H8, ^1^H9	–17.83	–20.6	5.4; −5.0	0.7; 0.2	–17.9
	^1^H12, ^1^H13	–27.70	–36.6	0.2; 0.5	0.8	–29.2
	^14^N11	14.88	21.8	–0.2	–0.4	17.6
	^14^N14	–6.08	–3.2	0.0	0.1	–7.6
	^17^O15, ^17^O16	–1.58	–1.2	9.2; −9.2	2.9; – 2.8	–2.1
benzyl	^13^C1, ^13^C2	23.06	1.7; −1.2	0.3; −1.2		22.9
*C*_2*v*_	^13^C3, ^13^C5	–21.84	1.2; −1.4	0.8; −0.5		–22.5
	^13^C4	23.75	0.0	0.0		23.8
	^13^C11	–42.86	–4.3	8.1		–50.4
	^13^C12	58.56	0.3	–0.6		63.6
	^1^H6, ^1^H7	–17.04	2.0; −2.3	1.4; −0.9		–15.9
	^1^H8, ^1^H10	8.63	0.8; −1.7	1.5; 0.2		6.1
	^1^H9	–19.28	2.0	–3.8		–19.5
	^1^H13, ^1^H14	–50.59	–1.2; −7.6	10.0; 6.6		–55.1
phenylaminyl	^13^C1	–25.12	–2.1	1.6		–27.7
*C_s_*	^13^C2	24.56	6.8	–1.3		25.6
	^13^C3	–43.66	–20.3	1.0		–50.8
	^13^C4	23.26	3.9	–0.4		24.1
	^13^C5	–25.36	0.9	2.0		–27.9
	^13^C6	28.42	–2.8	–2.4		30.4
	^1^H7	9.87	1.9	–4.3		7.9
	^1^H8	–19.60	–2.4	3.6		–19.5
	^1^H9	–19.96	–0.3	–2.4		–19.7
	^1^H10	10.04	–0.1	2.0		8.0
	^1^H11	–23.29	9.8	1.6		–24.8
	^1^H13	–41.98	–21.1	2.1		–45.6
	^14^N12	20.40	0.8	–3.7		24.5
1,3,2-benzodithiazolyl	^13^C1, ^13^C2	–4.99	15.8; −15.8	11.8		–7.1
*C*_2*v*_	^13^C3, ^13^C6	0.26	12.3; −12.3	–9.9		0.0
	^13^C4, ^13^C5	–0.27	–14.2; 14.2	3.8		–0.5
	^1^H7, ^1^H10	–1.46	–6.9; 6.9	6.0		–1.3
	^1^H8, ^1^H9	–1.20	8.7; −8.7	–1.1		–1.2
	^33^S11, ^33^S12	5.46	–13.7; 13.7	6.7		9.9
	^14^N13	28.39	0.0	–3.6		36.3
cyclo-hexyl	^13^C1	–0.96	1.3	1.8	1.1	–1.8
*C_s_*	^13^C2, ^13^C6	23.41	1.2; 13.4	18.9; 14.2	4.9; – 1.9	21.9
	^13^C3, ^13^C5	–25.36	0.8; −3.4	–6.2; – 4.5	0.2; 2.6	–33.1
	^13^C4	108.40	8.8	–41.7	44.6	119.9
	^1^H7, ^1^H13	17.57	2.3; −3.5	–1.4; 0.9	–2.6; 0.7	15.5
	^1^H8, ^1^H15	–22.56	–20.5; 0.8	0.5; 0.0	0.5; – 20.2	–23.1
	^1^H9, ^1^H16	0.52	–20.2; −21.6	–1.4; – 0.9	–1.2; – 0.4	–0.1
	^1^H10	0.16	–0.1	0.0	–0.1	–0.1
	^1^H11	2.48	–1.4	–1.8	–1.2	2.2
	^1^H12	–56.03	–11.9	–1.7	–20.0	–63.7
	^1^H14, ^1^H17	129.16	–46.0; 12.6	4.5; – 18.4	–8.4; – 41.3	117.5
1-adamantyl	^13^C1	–17.30	2.3	3.2	–6.7	–23.1
*C*_3*v*_	^13^C4	39.03	6.8	9.4	–26.5	38.8
	^13^C6	208.34	57.9	79.8	–139.4	223.4
	^13^C7	–17.30	2.3	4.2	–6.1	–23.1
	^13^C10	–4.12	0.0	0.0	0.0	–4.7
	^13^C13	39.03	7.0	18.1	–21.5	38.8
	^13^C15	–4.12	0.0	0.0	0.0	–4.7
	^13^C18	–4.12	0.0	0.0	0.0	–4.7
	^13^C21	–17.30	3.3	3.5	–6.1	–23.1
	^13^C24	39.03	15.0	12.2	–21.5	38.8
	^1^H2	19.64	3.5	2.2	1.5	17.5
	^1^H3	19.64	1.0	4.0	1.5	17.5
	^1^H5	11.72	–3.2	–4.3	9.8	10.9
	^1^H8	19.64	3.4	–2.5	–1.2	17.5
	^1^H9	19.64	0.9	–3.3	–2.7	17.5
	^1^H11	7.90	1.6	–4.0	6.9	6.7
	^1^H12	2.17	2.1	–0.4	0.6	1.9
	^1^H14	11.72	–3.2	–6.2	8.7	10.9
	^1^H16	2.17	–1.0	–1.4	–1.3	1.9
	^1^H17	7.90	–4.4	–6.0	3.3	6.7
	^1^H19	2.17	–1.0	1.9	0.6	1.9
	^1^H20	7.90	–4.3	0.2	6.9	6.7
	^1^H22	19.64	–3.4	2.5	–1.2	17.5
	^1^H23	19.64	–3.4	–0.1	–2.7	17.5
	^1^H25	11.72	–5.0	–5.0	8.7	10.9

a All reference values are CCSD/aug-cc-pVTZ-J results from ref (11).

**Figure 2:**
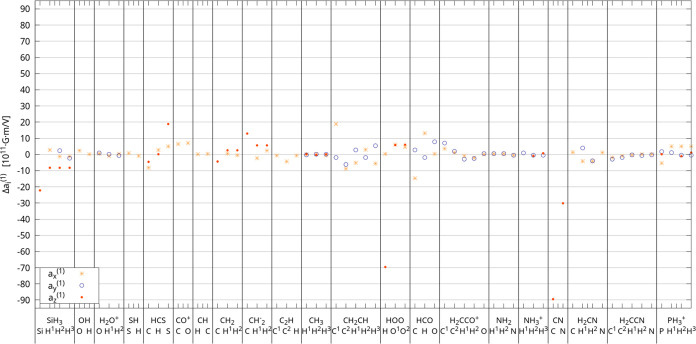
Absolute errors of *a_i_*^(1)^ for CAM-B3LYP. DLPNO-CCSD results used as a reference.

As can be seen from Table I, for CAM-B3LYP, the **A**_iso_ value is in good agreement with more advanced DLPNO-CCSD result with a difference of less than 30 MHz or ca. 5% of the DLPNO-CCSD value. Also, the small difference, less than 5%, between the first derivative of the Si nucleus is acceptable. The differences between CAM-B3LYP and DLPNO-CCSD for **A**_iso_ for hydrogen are larger, with DFT values almost 90% larger than the corresponding DLPNO-CCSD. However, the errors for the first derivatives for all hydrogen atoms are acceptable again, reaching about 14% for all the components. It is interesting to note that the results for the first derivatives for the *z*-component for both atoms for CAM-B3LYP are acceptable: about 5% error for Si and 16% for hydrogen atoms when compared to DLPNO-CCSD. The large differences between our results and Karna’s results for H atoms indicate the poor quality of his basis sets in the demanding core region of the hydrogen atoms. Table I also contains the results for the OH radical. We compare our results to the results of Karna,^20^ who used the uncorrelated Time Dependent UHF (TDUHF) method and the basis set of Sadlej^21^ unsuitable for the core regions.^20^ Predictably, his results are very far from our calculations; with the first derivative for H, his values are more than seven times larger than CAM-B3LYP or DLPNO-CCSD results.

The next system we analyze is the cation of aniline. The geometry of the cation and labeling of the atoms correspond to the geometry taken from ref (11) (see: Figure 1). Symmetry of the system (C_2v_) was used to present the results in a compact form of Table II.

It is evident from the presented results that the values of the **A**_iso_ hyperfine coupling tensor obtained in this work with CAM-B3LYP functional and DLPNO-CCSD method are very close to the benchmark CCSD values of Bartlett et al.^11^ We can also notice good agreement between DFT and DLPNO-CCSD for the *x-*components (along the *C*_2_ axis) for all atoms. The values of the Bloembergen effect constants *a_i_*^(1)^ obtained by CAM-B3LYP are in good agreement with the DLPNO-CCSD results. The largest difference occurs for the *x*-component for C2 and C4 atoms, almost 10 units (10^11^·G·m/V) or ca. 20%. The first derivatives with respect to the *x*-component are always the largest among the calculated derivatives.

Further results of our calculations are collected and displayed in Tables III and IV. As we already mentioned, the comparison of the first derivatives of **A**_iso_ obtained in this study to the previous studies is very limited. The comparison of our DLPNO-CCSD and CAM-B3LYP results for the **A** tensor itself to other results is also presented in Table III.

The data presented in Table III confirm the well-established fact that **A**_iso_ results span a wide range of values, e.g., for ^13^C isotope, **A**_iso_ varies from 20.74 MHz for CH_2_CH to 1529.90 MHz for CN at the DLPNO-CCSD level. We report only nonzero values of the components of *a_i_*^(1)^. The nonreported *a_i_*^(1)^ values are either exactly zero by symmetry or were neglected when very small (10^–2^ units or less).

As we already mentioned, the comparison of the first derivatives of **A**_iso_ obtained in this study to the previous studies is very limited. The performance of the proposed numerical approach can be indicated by comparing the results of CAM-B3LYP to DLPNO-CCSD.

Figure 2 displays the absolute error of the *a_i_*^(1)^ CAM-B3LYP results as compared to the DLPNO-CCSD values. For the majority of the studied systems, we notice a very good agreement (differences of less than 10 units) between both sets of results. The largest difference occurs for the **A**_iso_ derivatives for ^13^C in CN. The observed difference occurs also for the **A**_iso_ tensor itself.^10,30^ To accurately determine **A**_iso_ and its derivatives with respect to the electric field, a detailed study of the complete basis set limit at the CC level is needed. This lies beyond the scope of the present study.

## Conclusions

Although the effect of the external electric field on the hyperfine coupling tensor **A**_iso_ is seldom calculated for radicals in the gaseous phase, it can prove useful as a simple tool in analyzing the environmental effects on EPR experiments. This is the effect expressed as the first derivative of the **A**_iso_ tensor with respect to the components of the applied field, which can be simply calculated by numerical differentiation. Once an accurate quantum chemical method and robust, suitable basis sets are chosen, the technique proposed in this work allows a quick and reliable estimate of the effect. It can be of particular interest when validating the usefulness of new DFT functionals for analyzing EPR data. Some additional aspects need to be studied in more detail, mainly how to reach the complete basis set limit.

## Supporting Information Available

Structures of studied molecules and Cartesian coordinates of their geometries (PDF)
