# Supplementary material for: Electric Field Dependence of EPR Hyperfine Coupling Constants
Source: J Phys Chem A. 2024 Sep 12;128(38):8080–7. doi: 10.1021/acs.jpca.4c04480 (PMC11440590; doi:10.1021/acs.jpca.4c04480)
Supplement: Supplementary file 1 — jp4c04480_si_001.pdf [file jp4c04480_si_001.pdf]

Supporting Information for

Electric Field Dependence of EPR Hyperfine Coupling Constants

Tadeusz Pluta* and Grzegorz Skrzyński

Institute of Chemistry, University of Silesia in Katowice, Szkolna 9, 40-006, Katowice, Poland

*Corresponding author e-mail: tadeusz.pluta@gmail.com

Figure S1: Structure of 4-nitroaniline$^+$ with atom labels.

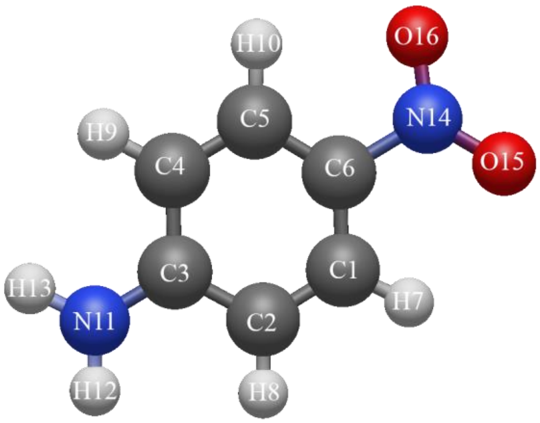

Figure S2: Structure of benzyl with atom labels.

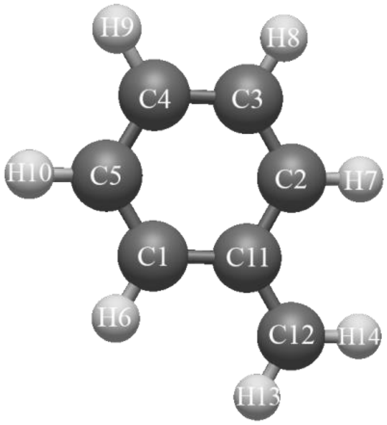

Figure S3: Structure of phenylaminyl with atom labels.

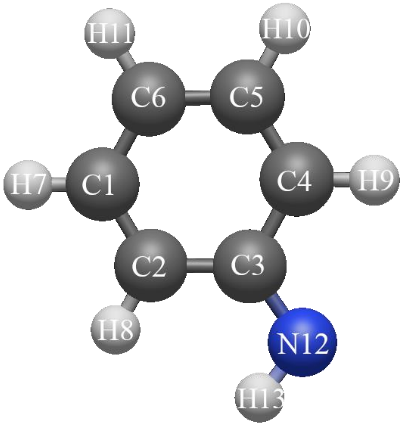

Figure S4: Structure of 1,3,2-benzodithiazolyl with atom labels.

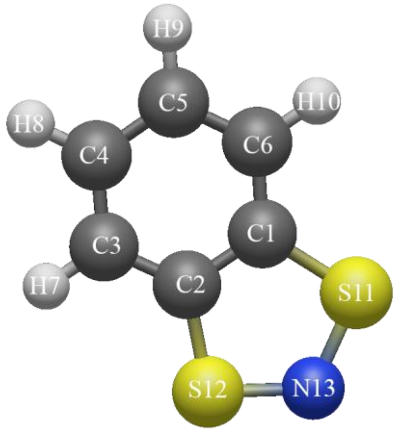

Figure S5: Structure of cyclo-hexyl with atom labels.

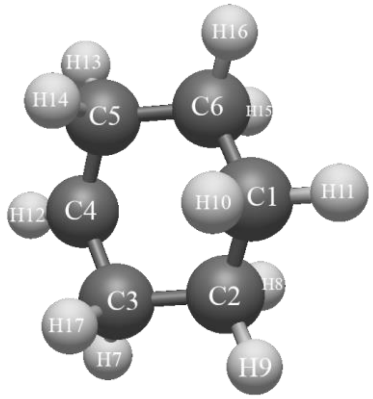

Figure S6: Structure 1-adamantyl with atom labels.

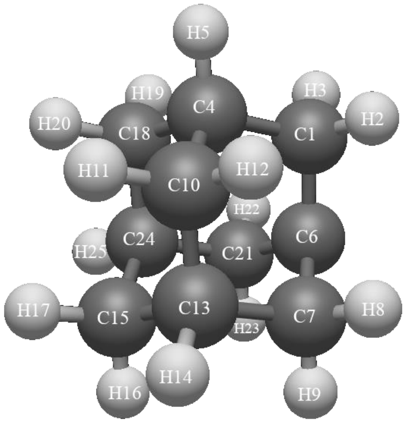

Table S1: Cartesian coordinates of studies molecules and cations (in Å). Geometry of SiH_3 taken from the Ref. [1], geometry of CN taken from the Ref. [2], while the rest of the geometries taken from the Ref. [3].

SiH_3

Si	0.000000	0.000000	0.000000
H	1.402474	0.000000	-0.455800
H	-0.701404	1.214868	-0.455800
H	-0.701404	-1.214868	-0.455795

OH

O	-1.218383	1.717364	0.000000
H	-0.246002	1.717364	0.000000

$Aniline^+$ cation

C	-0.256824	-1.198570	-0.015631
C	1.087091	-1.234821	0.057707
C	1.820065	-0.008497	0.131191
C	1.127138	1.243043	0.126439
C	-0.217181	1.254326	0.052411
C	-0.930492	0.040141	-0.019630
H	-0.828644	-2.113501	-0.071830
H	1.624269	-2.173735	0.062199
H	1.694350	2.162429	0.182472
H	-0.759109	2.188952	0.047516
H	-2.009653	0.059223	-0.078785
N	3.152119	-0.032051	0.204231
H	3.664096	-0.901911	0.209238
H	3.691911	0.819208	0.257001

H_2O^+ cation

O	1.130895	1.420494	-7.514301
H	2.132306	1.423751	-7.514301
H	0.799688	2.365552	-7.514301

SH

H	-0.236230	1.717364	0.000000
S	-1.578155	1.717364	0.000000

HCS

C	2.281734	0.918885	0.040780
H	3.369293	0.918885	0.027624
S	1.221886	0.918885	-1.103423

CO^+ cation

C	-1.165440	1.717364	0.000000
O	-0.048945	1.717364	0.000000

CH

H	-0.226858	1.717364	0.000000
C	-1.347527	1.717364	0.000000

CH_2

C	-5.234025	2.148289	-0.023721
H	-6.224911	2.148289	0.400104
H	-4.243140	2.148289	0.400104

CH_2^- anion

C	-5.234025	2.148289	-0.045004
H	-6.110269	2.148289	0.653956
H	-4.357781	2.148289	0.653956

C_2H

C	2.004321	0.918869	-0.000000
C	0.795099	0.918893	-0.000000
H	3.069006	0.918892	0.000000

CH_3

C	-3.837937	0.033754	0.350297
H	-3.328814	-0.864599	0.660118
H	-3.330837	0.753431	-0.271907
H	-4.854248	0.212281	0.662465

CH_2CH

C	-7.336618	0.087777	-2.583519
C	-6.036355	0.261057	-2.583505
H	-8.020372	0.936685	-2.583515
H	-7.785302	-0.899455	-2.583537
H	-5.348237	1.092059	-2.583489

HOO

H	3.091640	0.918885	-0.506692
O	1.330150	0.918885	-1.110419
O	2.239655	0.918885	-0.041027

HCO

C	0.000000	0.000000	0.000000
H	1.119250	0.000000	0.000000
O	-0.669245	0.972539	0.000000

H_2CCO^+ cation

C	0.000000	0.000000	0.000000
H	1.084600	0.000000	0.000000
H	-0.606960	0.899050	0.000000
C	-0.651940	-1.230300	-0.000000
O	-1.177870	-2.225760	0.000000

NH_2

H	2.132824	1.447192	-7.514301
H	0.821611	2.373865	-7.514301
N	1.108454	1.388740	-7.514301

NH_3^+ cation

N	-0.208872	-2.225354	-8.034806
H	-0.207212	-3.247164	-8.035082
H	-0.209701	-1.714697	-7.149749
H	-0.209703	-1.714198	-8.919571

CN

C	0.000000	0.000000	0.000000
N	0.000000	0.000000	1.171800

H_2CN

C	-3.319491	0.140709	1.439708
H	-3.885864	1.077465	1.439619
H	-3.885864	-0.796046	1.439658
N	-2.071425	0.140709	1.439623

H_2CCN

C	0.000000	0.000000	0.000000
H	1.078350	0.000000	0.000000
H	-0.546110	0.929870	0.000000
C	-0.691710	-1.208110	0.001190
N	-1.273320	-2.223770	0.002260

PH_3^+ cation

P	-0.398151	-2.225252	-8.034755
H	-0.028897	-3.571978	-8.034932
H	-0.029078	-1.552144	-6.868299
H	-0.029363	-1.552045	-9.201223

4-nitroaniline$^+$ cation

C	-0.246264	-1.219579	0.157178
C	1.100005	-1.244962	0.131299
C	1.830811	-0.009581	0.124322
C	1.130040	1.242883	0.147671
C	-0.216341	1.249630	0.178815
C	-0.894163	0.022936	0.182035
H	-0.840218	-2.121811	0.155146
H	1.642071	-2.180864	0.115383
H	1.694462	2.165582	0.140152
H	-0.787987	2.165772	0.205535
N	3.155008	-0.025383	0.096276
H	3.672741	-0.892838	0.078636
H	3.693567	0.829468	0.091537
N	-2.374140	0.040607	0.213384
O	-2.937453	-1.026147	-0.055379
O	-2.899933	1.120370	0.505221

benzyl

C	-3.254708	0.129429	-5.206971
C	-1.126934	1.255541	-5.168696
C	-1.771489	2.456666	-5.079962
C	-3.156211	2.512014	-5.053631
C	-3.887908	1.336563	-5.118041
H	-3.830772	-0.785770	-5.256992
H	-0.045222	1.217711	-5.188899
H	-1.196337	3.371647	-5.029916
H	-3.661757	3.464848	-4.983361
H	-4.968929	1.375025	-5.097797
C	-1.849640	0.049440	-5.235280
C	-1.190429	-1.193009	-5.327069
H	-1.747278	-2.115249	-5.377966
H	-0.113670	-1.250666	-5.348556

phenylaminyl

C	-0.241791	-1.169591	0.008258
C	1.121645	-1.179855	-0.038238
C	1.855737	0.034051	-0.050027
C	1.123706	1.247861	-0.011997
C	-0.238754	1.240842	0.034301
C	-0.933681	0.035701	0.044865
H	-0.789634	-2.102024	0.016756
H	1.665778	-2.115844	-0.066858
H	1.692228	2.166848	-0.021397
H	-0.784625	2.173874	0.062943
H	-2.013992	0.035517	0.081667
N	3.190200	0.118876	-0.094492
H	3.579493	-0.826492	-0.117945

1,3,2-benzodithiazolyl

C	-4.969124	0.005277	0.238661
C	-3.566188	0.005232	0.238539
C	-2.857467	1.213542	0.238610
C	-3.565967	2.409813	0.238802
C	-4.969191	2.409858	0.238923
C	-5.677767	1.213632	0.238854
H	-1.774965	1.214634	0.238517
H	-3.027934	3.348393	0.238858
H	-5.507165	3.348472	0.239072
H	-6.760269	1.214793	0.238947
S	-5.664044	-1.599252	0.238546
S	-2.871370	-1.599339	0.238304
N	-4.267734	-2.503846	0.238326

cyclo-hexyl

C	-0.807627	-2.651122	0.141999
C	0.714815	-2.747059	0.205056
C	1.350116	-1.357141	0.319003
C	0.724527	-0.558007	1.415285
C	-0.766321	-0.530698	1.507164
C	-1.362673	-1.935825	1.371341
H	2.427887	-1.440507	0.467546
H	0.997290	-3.340771	1.077936
H	1.103247	-3.264987	-0.673786
H	-1.097461	-2.099650	-0.757867
H	-1.245105	-3.647545	0.055807
H	1.297590	0.226822	1.890168
H	-1.087976	-0.067609	2.441343
H	-1.175264	0.093961	0.698929
H	-1.113302	-2.516619	2.262799
H	-2.451510	-1.876889	1.321820
H	1.212519	-0.838444	-0.641569

1-adamantyl

C	-0.662901	0.202345	0.553866
H	0.430223	0.203128	0.549758
H	-1.000598	1.242000	0.549897
C	-1.183871	-0.515346	1.823341
H	-0.812999	-0.004664	2.717212
C	-1.216645	-0.560164	-0.613762
C	-0.690186	-1.962827	-0.696141
H	0.402707	-1.980321	-0.710804
H	-1.047412	-2.472685	-1.594679
C	-0.690811	-1.967688	1.812777
H	-1.037981	-2.483095	2.713105
H	0.402720	-1.991479	1.830112
C	-1.211318	-2.693481	0.565852
H	-0.859936	-3.729760	0.566623
C	-2.744840	-2.665095	0.560782
H	-3.125623	-3.189430	-0.320555
H	-3.127822	-3.192686	1.439265
C	-2.717511	-0.496172	1.812965
H	-3.078692	0.536262	1.830417
H	-3.099999	-0.985921	2.713304
C	-2.713396	-0.493847	-0.695945
H	-3.068402	0.539930	-0.710454
H	-3.087715	-0.991283	-1.594488
C	-3.246635	-1.215715	0.566045
H	-4.340787	-1.202457	0.566958

References:

[1] Karna, S.P. Ab initio coupled Hartree–Fock study of the Bloembergen effect on paramagnetic systems: SiH_3 radical. *J. Comp. Chem.* **1999**, *20*, 1274-1280.

[2] NIST Computational Chemistry Comparison and Benchmark Database, NIST Standard Reference Database Number 101, Release 22, May 2022, Editor: Russell D. Johnson III. http://cccbdb.nist.gov/ (accessed 2024-07-03)

[3] Windom, Z.W.; Perera, A.; Bartlett, R.J. Benchmarking isotropic hyperfine coupling constants using (QTP) DFT functionals and coupled cluster theory. *J. Chem. Phys.*, **2022**, *156*, 094107.
